# Early Identification of Herbicide Modes of Action by the Use of Chlorophyll Fluorescence Measurements

**DOI:** 10.3390/plants9040529

**Published:** 2020-04-20

**Authors:** Sirous Hassannejad, Ramin Lotfi, Soheila P Ghafarbi, Abdallah Oukarroum, Amin Abbasi, Hazem M Kalaji, Anshu Rastogi

**Affiliations:** 1 Department of Plant Eco-Physiology, Faculty of Agriculture, University of Tabriz, Tabriz, Iran; sirous_hassannejad@tabrizu.ac.ir; 2 Dryland Agricultural Research Institute, Agricultural Research, Education and Extension Organization (AREEO), Maragheh, Iran; r.lotfi@areeo.ac.ir (R.L.); porheidarghafar@ut.ac.ir (S.P.G.); 3 University Mohammed VI Polytechnic (UM6P), Lot-660 Hay Moulay Rachid, Ben Guerir 43150, Morocco; abdallah.oukarroum@um6p.ma; 4 Department of Plant Production and Genetics, Faculty of Agriculture, Maragheh University, Maragheh, Iran; a.abbasi@maragheh.ac.ir; 5 Department of Plant Physiology, Institute of Biology, Faculty of Agriculture and Biology, Warsaw University of Life Sciences, 02-776 Warsaw, Poland; 6 Laboratory of Bioclimatology, Department of Ecology and Environmental Protection, Poznan University of Life Sciences, Piątkowska 94, 60-649 Poznan, Poland; anshu.rastogi@up.poznan.pl

**Keywords:** photosynthesis, photosynthetic efficiency, photosystem II (PSII), herbicide

## Abstract

The effect of seven herbicides (U-46 Combi Fluid, Cruz, MR, Basagran Bromicide, Lumax, and Gramoxone) on *Xanthium strumarium* plants was studied. Chlorophyll content and fluorescence, leaf temperature, and stomatal conductance were evaluated at 12 h, 36 h, 60 h, and 84 h after herbicides application. U46 Combi Fluid, Cruz, and MR did not have a significant effect on chlorophyll fluorescence induction curves as compared to the control treatment. However, Basagran, Bromicide, Lumax, and Gramoxone showed significant changes in the shape of polyphasic fluorescence transients (OJIP transients). Variations in chlorophyll content index, leaf temperature, and stomatal conductance parameters were dependent on the type of applied herbicide. Our study revealed that the specific impact of the applied herbicides on the photosynthetic efficiency of plants is related to their chemical groups and their mechanism of action.

## 1. Introduction

In agriculture, herbicides are widely used to reduce the predominance of nondesirable plant species. Some of them act solely via contact to the plants, whereas some others penetrate the vascular system, causing damage to plants. They cause severe damage to nucleic acids in plants and modify the plastid metabolism and cell cycle [1,2].

Herbicides have a strong impact on photosynthetic organisms and their effects depend on their mode of action, dose, and plant species [3]. Some studies have indicated that the application of herbicides have a negative influence on plant pigment content and have reported the destruction of chloroplasts and thylakoids [4]. Herbicides were observed to have an impact either on one or both phases (photoinduced electron transport and dark reactions) of photosynthesis [5]. In photosystem II (PSII), the acceptor side is a major location for the inhibition of photosynthetic electron transport. The inhibition mechanism can be explained by the competitive binding of the D_1_ protein of quinone B (Q_B_^−^) with the herbicides [6], thus preventing the reoxidation of quinone A (Q_A_^−^) by forwarding electron transfer. Several commercially available herbicides have been observed to inhibit PSII electron transport in higher plants. For instance, Armel et al. [7] have suggested that PSII-inhibiting herbicides interrupt electron flow by binding to the D_1_ protein at the plastoquinone B (PQB).

During photosynthesis, part of the solar radiation absorbed by plants is reemitted in the form of fluorescence. Therefore, by mathematical calculations, it has been established that photosynthetic processes can be monitored by measuring chlorophyll fluorescence (ChlF) signals from plants [8,9], including the impact of herbicides [10,11]. Under nonstress conditions, light energy is absorbed by chlorophyll and used in photochemical processes. However, a proportion of this energy is released in the form of heat and fluorescence. When the electron transport in the photosynthetic system is partially or completely blocked due to a stress factor, the emitted fluorescence intensity increases, reflecting less use of photosynthetically active radiation by plants [12,13,14,15,16,17].

When dark-adapted photosynthetic samples are illuminated by saturated light, polyphasic fluorescence transients (OJIP transients) are observed and can be plotted on a logarithmic time scale [18]. The rise in the fluorescence transients is associated with the reduction of electron carriers in the thylakoid membrane. The shape of the OJIP curves under the existence of any type of stress could change according to the photosynthetic efficiency of plants. The analysis of chlorophyll fluorescence parameters offers a rapid and precise technique for the purpose to detect and quantify stress tolerance in plants [19]. Strasser et al. [20] have shown the significance of the JIP-test for the purpose of understanding different changes in PSII photochemistry caused by different stress factors. Strasser et al. [21] found that the analysis of chlorophyll fluorescence kinetics and fluorescence parameters calculated by the JIP-test provides detailed information about the functioning of the photosynthetic apparatus. Therefore, with analyzing changes in chlorophyll fluorescence, not only we can determine the location of the electron transport inhibition by herbicides, but this method can also evaluate the damage done by herbicides. Thus, through this research, we monitored the changes in the OJIP transient and ChlF parameters for the purpose ofidentifying the different herbicides modes of action in *Xanthium strumarium*.

## 2. Materials and Methods

### 2.1. Plant Materials, Growth Conditions, and Herbicide Treatments

The effect of seven herbicides (U-46 Combi Fluid, Basagran, Bromicide, Cruz, Mister (MR), Lumax, and Gramoxone) on photosynthetic performance of the common cocklebur (*Xanthium strumarium*) was studied. U-46 Combi Fluid, Basagran, Cruz, MR, and Gramoxone were selected as they are most common herbicides used in Iran, whereas Lumax (Mesotrione + Metolachlor + Terbuthylazine) and Bromicide (Bromoxynil + MCPA) are novel and combined herbicides, and were selected for this study because of their novelty. The experiment was based on the randomized complete block (RCB) design with three replications performed in 2015 under greenhouse conditions (Tabriz, Iran). For the investigation, 10 seeds of *Xanthium strumarium* were planted at 1-cm depth in each plastic pot (20 cm × 20 cm), with 1.0 kg of perlite, then irrigated to reach 100% of the field capacity. The pots were placed in a greenhouse under natural light exposure and a minimum and maximum temperatures of 27 °C and 30 °C, respectively. After the seedling establishment phase, the plants were thinned to five plants per pot. During the growth period, the pots were weighted, and weight loss was adjusted with Hoagland solution (pH 6.5 to pH 7 and Electrical conductivity of 1.3 dS m^−1^) [22]. Every 20 days, the perlite in the pots was washed to prevent a further increase in electrical conductivity due to the addition of the Hoagland solution. Herbicides were applied on 30 days after the emergence of plants following the recommended dose in the field (2 L ha^−1^). A mock application with distilled water was applied as a control.

### 2.2. Measurement of Chlorophyll a Fluorescence

A Handy-PEA fluorometer (Hansatech Instruments Ltd., UK) was used to measure ChlF signals and plotting the induction curves (OJIP transient). Before the measurement, the plants were dark-adapted for a minimum of 30 min. The measurement was performed at a fully developed leaf adaxial side at 12 h, 36 h, 60 h and 84 h post-herbicide application. On the base of the recorded OJIP transient during the 1-s period, several expressions and fluorescence parameters describing the physiological state of the photosynthetic sample were calculated. Relative variable fluorescence (Vt) was calculated as Vt = (F_T_ − F_O_)/(F_M_ − F_O_). Some basic and important chlorophyll a fluorescence parameters were: Fo and Fm, the initial and maximum fluorescence, respectively; PIabs, the performance index or photosynthesis relative vitality; Tfm, the time taken to reach F_M_, an indicator of Q_A_ reduction rate of the PSII acceptor, i.e., the rate of PSII electron transport; Fv, the variable chlorophyll fluorescence (F_V_ = F_M_ − Fo); Fv/Fo, the activity of the water-splitting complex on the donor site of the PSII (F_V_/Fo = (F_M_ − Fo)/Fo); Fv/Fm, the maximum quantum yield of PSII (F_V_/F_M_ = (F_M_ − Fo)/F_M_); and Sm, the energy necessary for the closure of all the reaction centers.

### 2.3. Stomatal Conductance, Chlorophyll Content Index, and Leaf Temperature

The leaf temperature (LT) (°C) and chlorophyll content index (CCI) was monitored with an infrared thermometer (TES-1327) and a chlorophyll meter CCM-200 (Opti-Science, USA), respectively. Stomatal conductance was measured with an AP4 porometer (Delta-T Devices, Cambridge, UK). The fully expanded leaves free from any physical stress and disease were selected. The measurement was on the abaxial leaf surfaces. Three replicates from each sample were measured and the mean value was used to represent the stomatal conductance. All of these physiological parameters were measured 84 h after herbicides application.

### 2.4. Statistical Analysis

Data were analyzed using SAS 9.1 software (SAS Institute, USA). Means comparison was performed according to the LSD (least significant differences) test at *p* ≤ 0.05 and *p* ≤ 0.01.

## 3. Results

### 3.1. Chlorophyll and Fluorescence Induction Curves

**Figure 1:**
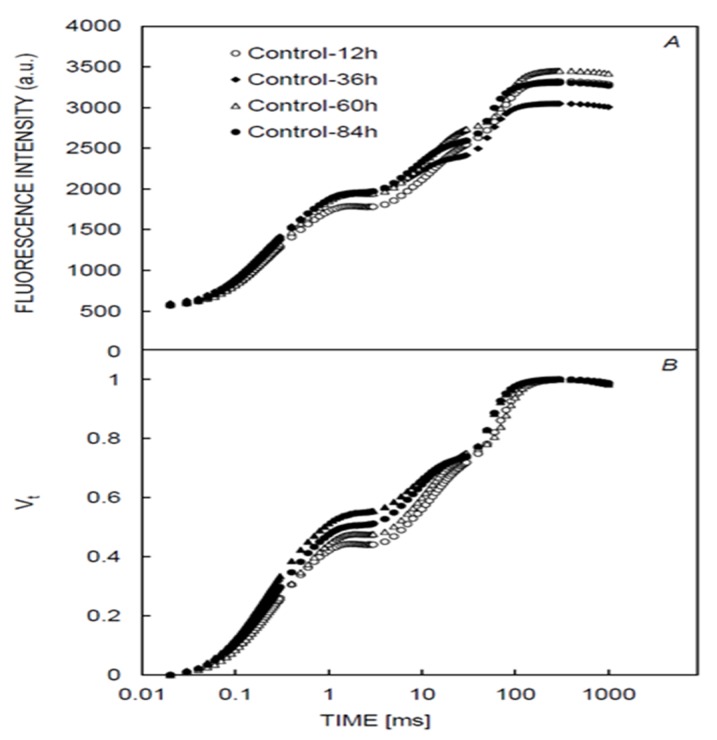
Chlorophyll *a* fluorescence from control. Samples which showed the typical polyphasic fluorescence (OJIP) curve at different time intervals (**A**) were plotted as fluorescence intensity, whereas (**B**) was plotted as relative variable fluorescence, Vt.

Chlorophyll fluorescence measurements of the control samples showed the typical polyphasic rise OJIP transient. The O step (F_20μs_) recorded at all measurement times was constant. However, at the J step, there was a significant difference between the various times of measurement observed when the curves were plotted as the relative variable fluorescence (Vt) (Figure 1).

**Figure 2:**
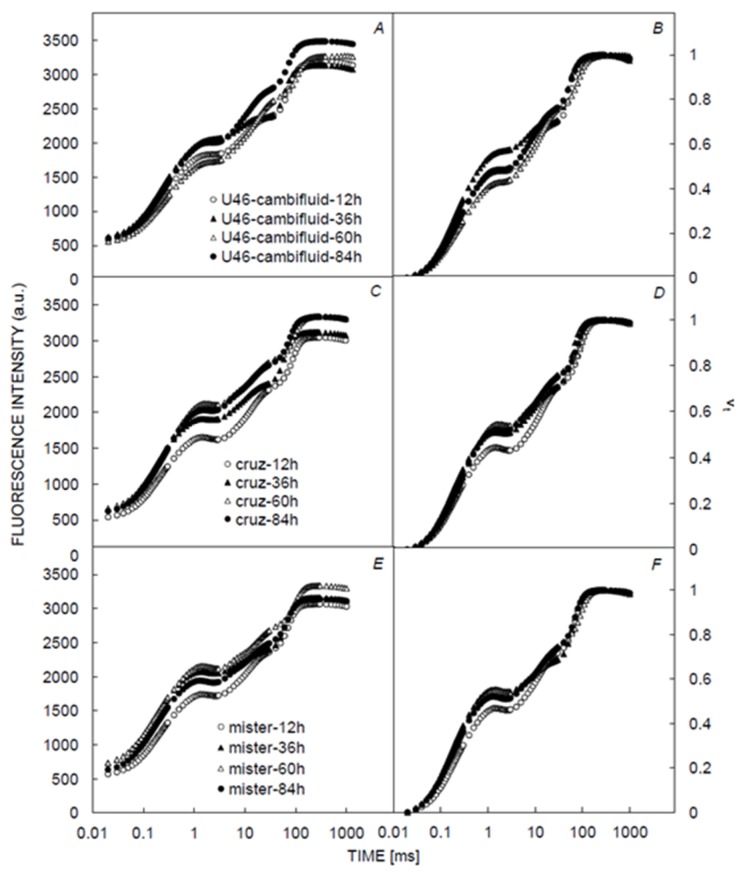
Effect of herbicides on chlorophyll a fluorescence. Effects of different herbicides on the OJIP curve are shown as fluorescence intensity (**A**,**C**,**E**) and relative variable fluorescence (**B**,**D**,**F**).

The application of U46Combi Fluid, Cruz, and MR on *Xanthium strumarium* plants did not show any effect on the shape of OJIP transient curves after 12 h, 36 h, 60 h, and 84 h treatments when compared to the control. Similar to the controlled conditions, the fluorescence level at the J step (F_J_) showed a significant difference between the different times of measurements when the curves were plotted as relative variable fluorescence Vt (Figure 2).

**Figure 3:**
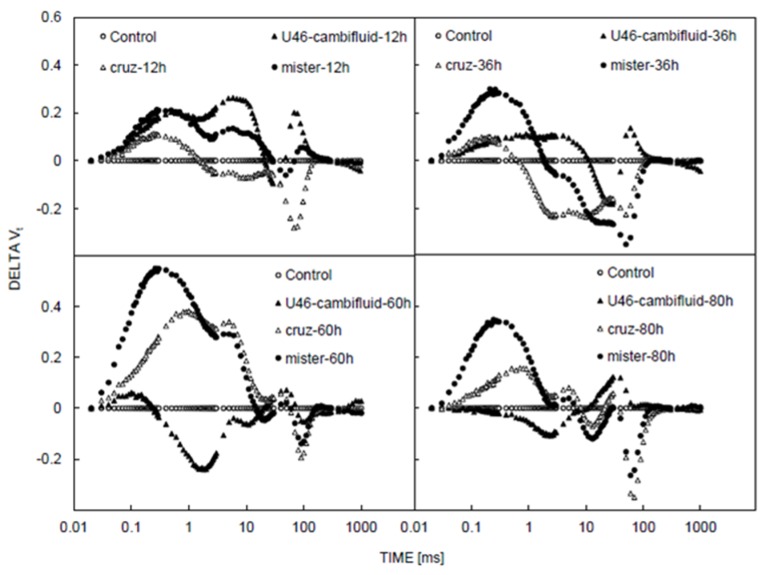
Effect of herbicides on OJIP fluorescence rise kinetics. Effects of different herbicides on OJIP fluorescence rise kinetics analyzed by calculating the difference in the variable fluorescence curves (ΔVt) at different time intervals.

Changes in OJIP fluorescence rise kinetics after U46 Combi Fluid, Cruz, and MR treatments were also analyzed by calculating the difference in the variable fluorescence curves (ΔV_t_) (Figure 3). ΔV_t_ curves were constructed by subtracting the normalized fluorescence values (between O and P) recorded in treated plants from those recorded in the control plants at the same time of treatment. This analysis showed the appearance of three bands, ΔK (at 300 μs), ΔJ peak (at 2 ms), and ΔI (at 10–30 ms). The change was observed to be more significant after 60 h of treatment.

**Figure 4:**
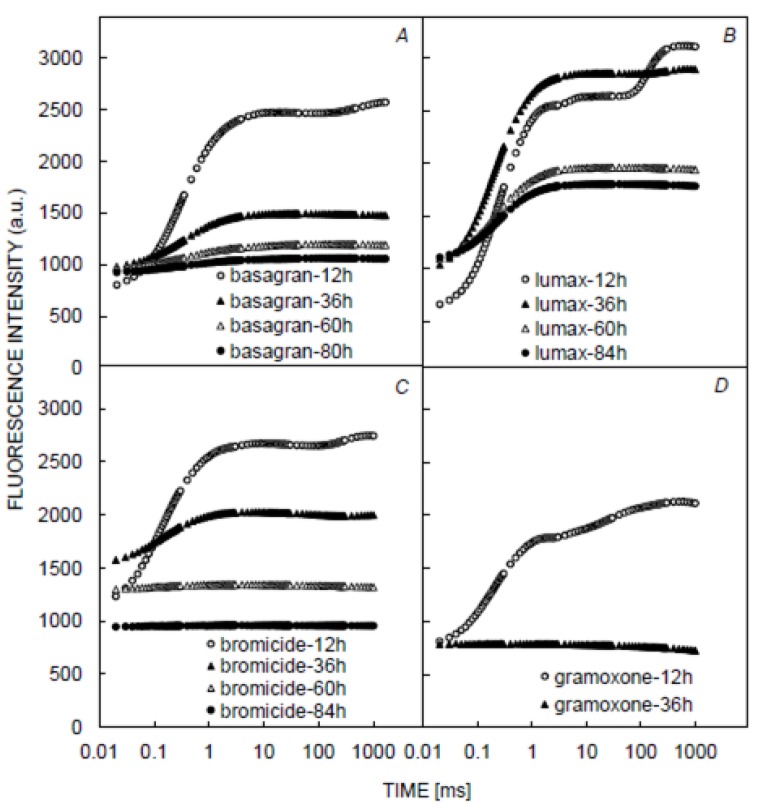
Effect of different herbicides on the shape of OJIP transients. Figures shows the effect of *Basagran* (**A**)*, Bromicide* (**B**)*, Lumax* (**C**), and *Gramoxone* (**D**) on the shape of OJIP curve.

The application of Basagran, Bromicide, Lumax, and Gramoxone herbicides significantly changed the shape of OJIP transients (Figure 4). Indeed, the J-I and I-P phases of the OJIP curve were not expressed, and at J step, the fluorescence was observed to be close to F_M__,_ except in the OJIP transient recorded in plants treated with Gramoxone and Lumax treatments at 12 h.

### 3.2. Chlorophyll and Fluorescence Parameters

In comparison with control plants, the initial fluorescence (F_O_) was increased in all herbicides treated plants. On the contrary, maximum fluorescence (F_M_), variable fluorescence (F_V_), the relative contribution of the trapping flux to the total deexcitation fluxes of excited chlorophyll (F_V_/F_O_), the maximum quantum efficiency of PSII (F_V_/ F_M_), and the performance index (PI_ABS_) was observed to be decreased in herbicides treated plants. With increasing times of measurement, the effects of herbicides on ChlF parameters were observed to be more evident. Among the studied herbicides, Bromicide, Lumax, and Gramoxone showed significant impacts on studied ChlF parameters. The time needed to reach F_M_ (T_FM_) increased at 12 h in most cases, but later on (at 36 h, 60 h, and 84 h following application), it decreased. The application of U-46 Combi Fluid at 60 h, as well as Cruz and MR, increased the average redox state of Q_A_ in the time from 0 to T_FM_ (Tfm). At the same time, the average fraction of open reaction centers was observed to be reduced with the application of Basagran, Bromicide, Lumax, and Gramoxone (Table 1).

### 3.3. Chlorophyll Content Index, Leaf Temperature, and Stomatal Conductance (gs)

Table 1 shows that the chlorophyll content index of plants decreased when different herbicides were applied, except for U-46 Combi Fluid. In most times after herbicide application, U-46 Combi Fluid and Cruz significantly decreased leaf temperature and increased stomatal conductance (*g*_s_), but MR, Basagran, Bromicide, Lumax, and Gramoxone enhanced LT and reduced the *g*_s_ of plants. These findings revealed that Lumax, and especially Gramoxone, had the greatest effect on the reduction of CCI and *g*_s_ compared to the rest of the evaluated herbicides (Table 1). The closure of stomata may be responsible for the observed increase in LT.

## 4. Discussion

The effects of herbicides on plants depend on their particular location, where a physiological reaction has been inhibited in the plant cell and its compartments. In the chloroplast, two main sites might be the target for herbicide actions. The first target site in the photosynthetic apparatus is represented by the electron transport chain (ETC), which is involved in phosphorylation and NADP photoreduction. Another leading target site is the biosynthesis of chlorophylls and carotenoids, which are contained in the light-harvesting complex (LHC) and the antennae of the photosynthetic reaction centers [23]. In our research, we observed the changes in both target sites after the application of different herbicides.

The OJIP transient recorded in *Xanthium strumarium* plants treated with Cruz, MR and *U*-*46* *Combi Fluid* herbicides were observed to keep the normal pattern and were polyphasic like the control treatment (Figure 1 and Figure 2). At 60 h of exposure time, some of the studied herbicides (Cruz, MR, and *U*-*46* *Combi Fluid*) were observed to affect the oxygen-evolving complex (appearance of K band could be related to inactivation of the OEC, i.e., the PSII donor side [24]) and inactivated the ferredoxin-NADP oxidoreductase (FNR) (Figure 3). However, leaves sprayed with Basagran, Bromicide, Lumax, and Gramoxone herbicides significantly affected the shape of the OJIP curves, even at low exposure time (Figure 4). The observation was found to be similar to DCMU treatment [25]. In other words, it can be said that the mode of action of Basagran, Bromicide, Lumax, and Gramoxone treatments was through the inhibition of electron transfer between Q_A_ and Q_B_ (the primary and secondary quinones) of PSII [26,27,28]. It has been previously reported that the photosynthetic electron transport chain gets interrupted due to herbicide exposure, leading to the concomitant inhibition of adenosine triphosphate (ATP) production and carbon fixation [27,29]. For four herbicides, it has been observed that, by increasing the times of measurement (36 h, 60 h, and 84 h), the OJIP curves were suppressed and turned into nearly straight lines. It has been observed that the effect of Gramoxone herbicide was significantly different than the effects of other herbicides on OJIP transients. Indeed, Gramoxone caused a total inhibition of photosynthetic electron transport (Figure 4). In agreement with the theory of Duysens and Sweers [30], the inflections of the ChlF induction curve reflect changes in the net reduction rate of Q_A_, which depend on the kinetics of the redox reactions between various components of the photosynthetic electron transport. This allows the use of the OJIP transient as a quick monitor of both the electron donor and the electron acceptor sides, and, according to this theory, the critical point of each herbicide on these processes [31].

The observations found in this work are in accordance with previous studies suggesting that U-46 Combi Fluid caused growth inhibition and increased the biosynthesis of abscisic acid, the stomata closure, and the restriction of CO_2_ diffusion through the stomata, indicating the accumulation of reactive oxygen species (ROS) [32,33]. Chiang et al. [34] reported that under the light-illuminated condition, Gramoxone (also known as paraquat or methyl viologen), accepts electrons directly from the FeS-clusters of PSI and creates a radical anion that is reoxidized by molecular oxygen. Therefore, it produces ROS directly in the form of superoxide anion radicle and H_2_O_2_ as a secondary product, resulting into the damadge of photosynthetic structure in thylakoids. The blockage of electron transfer is not only due to damage by ROS, but also by the effective removal of NADPH from the metabolism which in turn starves the Calvin circle [35]. Furthermore, the Gramoxone was reported to first affect the PSI, and was then observed to damage the surrounding area along with PSII [36]. The authors proposed that the electron leakage in the thylakoid membrane and the damage to the thylakoid membrane due to its oxidation in chloroplasts interrupts the electron transfer from PSII to photosystem I (PSI). This interruption of photosynthetic electron transport changes the shape of the OJIP curves [23].

The study clearly showed that Basagran, Bromicide, Lumax, and Gramoxone herbicides significantly reduced the CCI and *gs*, in addition to an increase in leaf temperature, in comparison to the control (Table 1). Duke & Dayan [37] have shown that the primary herbicide targets the chlorophyll biosynthesis in the chloroplast by acting on protoporphyrinogen oxidase (PPO), which catalyzes the conversion of protoporphyrinogen to protoporphyrin. The process involves the unregulated accumulation of the highly photodynamic pigment protoporphyrin, which causes rapid membrane peroxidation when exposed to light.

In the present work, the application of Basagran, Bromicide, Lumax, and Gramoxone significantly influenced the PSII activity of *Xanthium Strumarium* plants when compared with U-46 Combi Fluid, Cruz, and MR. The herbicides Basagran, Bromicide, Lumax, and Gramoxone also enhanced the F_O_ and decreased the F_M_ of the plants (Table 1). F_O_ is the fluorescence level when Q_A_ is oxidized, and it may change when exposed to stress [38]. An increase in F_O_ indicates a decrease in the rate constant of energy trapped by PSII reaction centers [39], which may be the result of damage to PSII core unit, observed in several previous studies [40]. The increase of F_O_ was also reported to be related to an accumulation of reduced plastoquinone (PQ). This accumulation was found to induce LHCII phosphorylation as a result of its exposure to environmental stresses [24]. As a result of herbicide application, the reduction in F_M_ may appear. The process is very common and appears together with photoinhibition, even before significant reduction of the total amount of photosystems takes place. Therefore, the decrease in F_M_ may be due to the inhibition of electron transfer at PSII donor side, which results in T_FM_ decreases at most of the exposure times of measurement (Table 1).

The application of U46Combi Fluid, Cruz, and MR on *Xanthium strumarium* plants provoked the appearance of the band at K step, which was related to damage to the oxygen-evolving complex [24], the appearance of the band’s J step, linked to an accumulation of Q_A_-, and the appearance of the band’s I step, associated with the inactivation of ferredoxin-NADP oxidoreductase (FNR) [8].

Also, the application of Basagran, Bromicide, Lumax, and Gramoxone, in relationship to other herbicides, destroyed the PSII reaction centers in treated plants (photochemically active). Thus, the electron transport capacity in PSII and the quanta absorbed per unit time decreased (Table 1).

Fluorescence parameter F_V_/F_O_ was observed to be a sensitive component in the photosynthetic electron transport chain. A decrease in this ratio (Table 1) as a consequence of herbicide application was observed to be a result of photosynthetic electron transport destruction, which affects the average redox state of Q_A_ in plants (in the time interval of 0 to T_FM_). The observed decrease of the S_M_ may be due to the inhibition of electron transport at the donor site of the PSII [41].

The most popular parameter of the JIP-test is the performance index (PI_ABS_). The photosynthetic performance index (PI_ABS_) is an indicator of photosynthesis vitality. The application of Basagran, Bromicide, Lumax, and Gramoxone, in comparison with other herbicides, impaired the light reactions of photosynthesis as a result of the reduction in PI_ABS_ (Table 1). A decrease in PI_ABS_ may be related to the effects of those herbicides upon the density of the reaction centers of PSII antenna chlorophyll, the maximum quantum yield for primary photochemistry, and the quantum yield for electron transport.

Based on the results, the study confirmed that the application of chlorophyll fluorescence measurements and the analyses of fluorescence parameters by JIP-test would identify their specific effect on photosynthetic electron transport.

## Figures and Tables

**Table 1:** Changes in chlorophyll content index (CCI), leaf temperature (LT), stomatal conductance (*g*_s_), minimal fluorescence (Fo), maximal fluorescence (Fm), variable fluorescence (Fv), water-splitting complex on the donor site of the PSII (Fv/Fo), maximum quantum yield of PSII (Fv/Fm), photosynthesis relative vitality (PI), Q_A_ reduction rate of the PSII acceptor (Tfm), and energy necessary for the closure of all the reaction centers (Sm) in response to different herbicides. Data are presented as mean from at least three sets of different measurements (least significant differences (LSD 5% and 1%) of means mentioned below the table).

	CCI	LT	gs	Fm	F0	Fv	Fv/Fm	Fv/Fo	PI	Sm	**Tfm**
**Control**											
12	17.8	17.5	1.45	3319	492	2827	0.85	5.75	2.37	22.99	300
36	19.3	18.2	0.52	3047	511	2536	0.83	4.96	1.27	16.88	270
60	16.7	23.7	0.59	3450	486	2964	0.86	6.10	2.35	22.54	290
84	17.7	29.7	0.15	3304	484	2820	0.85	5.83	1.87	17.23	290
**U.46 CombiFluid**											
12	18.2	15.9	1.15	3188	501	2687	0.84	5.36	1.80	20.10	250
36	18.8	13.6	1.38	3135	546	2589	0.83	4.74	1.10	16.07	240
60	19.1	21.7	0.43	3271	491	2780	0.85	5.66	2.47	24.17	700
84	19.3	29.4	0.38	3884	519	3365	0.85	6.48	1.93	14.50	270
**Cruz**											
12	16	17.1	0.88	3050	464	2586	0.85	5.57	2.13	26.37	290
36	15.5	15.9	1.75	3123	572	2551	0.82	4.46	1.18	19.44	230
60	15.4	22.3	1.28	3342	585	2757	0.83	4.71	1.29	23.65	290
84	14.1	29.2	1.23	3236	536	2700	0.84	5.22	1.49	20.71	290
**Mister**											
12	15.9	19.7	0.34	3065	492	2573	0.84	5.23	1.76	21.84	270
36	15	18.6	0.35	3157	665	2492	0.79	3.75	0.80	21.03	280
60	13.1	20.3	0.42	3334	675	2659	0.80	3.94	0.92	23.47	280
84	13.8	30.4	0.16	3139	559	2580	0.82	4.62	1.17	19.53	260
**Basagran**											
12	14.6	17.3	0.1667	2569	741	1828	0.71	2.47	0.10	20.02	900
36	14	22.2	0.42	1493	978	515	0.35	0.85	0.01	0.78	100
60	13.7	33	0.32	1196	968	228	0.19	0.75	0.01	1.75	100
84	11	27.5	0.1667	1061	926	135	0.13	0.72	0.00	1.48	80
**Bromicide**											
12	14.2	18.2	0.28	2746	1231	1515	0.55	1.23	0.02	17.95	900
36	14.8	18.2	0.1733	2028	1583	445	0.22	0.76	0.00	0.13	7
60	14.6	21.8	0.1513	1344	1304	40	0.03	0.68	0.00	0.13	5
84	12.9	30.2	0.1643	963	949	14.67	0.02	0.68	0.00	0.13	5
**Lumax**											
12	13.1	19.7	0.1497	3120	552	2568	0.82	4.65	0.45	28.66	700
36	12.2	19	0.36	2898	972	1926	0.67	1.98	0.03	6.33	800
60	12.8	21.1	0.38	1962	1076	886	0.45	0.95	0.01	0.45	28
84	12.5	30.3	0.1543	1804	1092	712	0.40	0.89	0.01	0.28	27
**Gramoxone**											
12	17.2	19.8	0.17	2128	775	1353	0.64	1.75	0.14	13.60	600
36	3.9	23.3	0.66	786	788	15	0.37	0.68	0.00	0.13	4
60	0	0	0	0	0	0	0.00	0.00	0.00	0.00	0
84	0	0	0	0	0	0	0.00	0.00	0.00	0.00	0
**LSD 5%**	0.9	0.493	0.124	21.4	9.05	6.16	0.032	0.773	0.008	0.152	4.17
**LSD 1%**	1.2	0.659	0.166	28.6	12.09	8.23	0.043	1.03	0.001	0.205	5.6
